# Stereotactic Body Radiotherapy as Treatment for Organ Confined Low- and Intermediate-Risk Prostate Carcinoma, a 7-Year Study

**DOI:** 10.3389/fonc.2014.00240

**Published:** 2014-09-02

**Authors:** Alan Jay Katz, Josephine Kang

**Affiliations:** ^1^Flushing Radiation Oncology Services, Flushing, NY, USA; ^2^Department of Medicine, NYU Langone Medical Center, New York, NY, USA

**Keywords:** prostate cancer, stereotactic radiotherapy

## Abstract

**Objectives:** Stereotactic body radiation therapy (SBRT) takes advantage of the prostate’s low α/β ratio to deliver a large radiation dose in few fractions. Initial studies on small groups of low-risk patients support SBRT’s potential for clinical efficacy while limiting treatment-related morbidity and maintained quality of life. This prospective study expands upon prior studies to further evaluate SBRT efficacy for a large patient population with organ confined, low- and intermediate-risk prostate cancer patients.

**Methods:** Four hundred seventy-seven patients with prostate cancer received CyberKnife SBRT. The median age was 68.6 years and the median PSA was 5.3 ng/mL. Three hundred twenty-four patients were low-risk (PSA <10 ng/mL and Gleason <7), 153 were intermediate-risk (PSA 10–20 ng/mL or Gleason = 7). Androgen deprivation therapy was administered to 51 patients for up to 6 months. One hundred fifty-four patients received 35 Gy delivered in five daily fractions; the remaining patients received a total dose of 36.25 Gy in five daily fractions. Biochemical failure was assessed using the phoenix criterion.

**Results:** Median follow-up was 72 months. The median PSA at 7 years was 0.11 ng/mL. Biochemical failures occurred for 11 low-risk patients (2 locally), 14 intermediate-risk patients (3 locally). The actuarial 7-year freedom from biochemical failure was 95.6 and 89.6% for low- and intermediate-risk groups, respectively (*p* < 0.012). Among patients with intermediate-risk disease, those considered to have low intermediate-risk (Gleason 6 with PSA >10, or Gleason 3 + 4 with PSA <10; *n* = 106) had a significantly higher bDFS than patients with high intermediate-risk (Gleason 3 + 4 with PSA 10–20 or Gleason 4 + 3; *n* = 47), with bDFS of 93.5 vs. 79.3%, respectively. For the low-risk and low intermediate-risk groups, there was no difference in median PSA nadir or biochemical disease control between doses of 35 and 36.25 Gy.

**Conclusion:** CyberKnife SBRT produces excellent biochemical control rates. Median PSA levels compare favorably with other radiation modalities and strongly suggest durability of response. These results also strongly suggest that 35 Gy is as effective as 36.25 Gy for low- and intermediate-risk patients.

## Introduction

Since the first report of a highly hypofractionated regimen for prostate cancer was published over 20 years ago (1), many additional reports documenting the use of five-fraction dose schemes utilizing special image-guided technology, termed stereotactic body radiotherapy (SBRT), have appeared (2–6), with excellent outcomes. Biochemical disease-free survival (bDFS) for low-risk prostate cancer is greater than 90% for SBRT. These results are consistent with radiobiological data suggesting that prostate cancer is especially responsive to changes in dose per fraction, with α/β ratio for prostate cancer is approximately 1.5 (7, 8). SBRT has potential to increase the number of patients worldwide who can access treatment, due to the marked decrease in the number of fractions required. Cost and patient convenience can also be favorably impacted (9).

Up until recently, excellent early results have been met with some skepticism over the durability of these early results. This has led to reluctance on the part of the radiotherapy community to embrace this treatment method, in spite of recent support of this treatment by ASTRO and NCCN (10, 11). To address this concern, we present the biochemical outcomes of a large cohort of low- and intermediate-risk patients treated with Cyberknife SBRT with as long as 8-year follow-up. Toxicity and quality of life (QOL) data on these patients will be presented in greater detail separately.

## Materials and Methods

### Patient selection

The study cohort was composed of 477 men with biopsy-proven, newly diagnosed non-metastatic prostate cancer, treated between early 2006 and January 2010 at Winthrop University Hospital. Data were analyzed for all clinically localized, low- or intermediate-risk prostate cancer patients, treated with CyberKnife SBRT. The treatment protocol was IRB-approved and the first 15 patients were treated in a prospective fashion to assess the feasibility of the approach in our hands. Subsequent patients were treated according to this approved protocol, but not as part of a prospective study. All patients provided informed consent for their outcomes to be incorporated in this retrospective study.

For the purposes of this analysis, we include only the low- and intermediate-risk patients, following standard D’Amico risk stratification (low-risk: PSA <10 and Gleason sum of 6 and clinical stage T1c–T2a, intermediate-risk: PSA 10–20 or Gleason sum of 7 or clinical stage T2b).

### Treatment

Fiducial-based image-guided SBRT was delivered using the CyberKnife system (Accuray Inc., Sunnyvale, CA, USA). The treatment specifics of Cyberknife have been published previously (12). General techniques are briefly outlined here. Four gold fiducials were placed in the prostate trans-perineally with ultrasound guidance. This was followed by a non-contrast CT scan in the supine position and in an alpha cradle. Except for those patients that could not undergo an MRI scan, MRI images were obtained and fused into the CT images to better visualize the inferior portion of the prostate. No catheter was used. Anatomical contours of the prostate, seminal vesicles, rectum, bladder, penile bulb, femoral heads, and testes were generated. With homogeneous planning, dose was prescribed to the planning target volume (PTV) that consisted of a volumetric expansion of the prostate by 5 mm, reduced to 3 mm in the posterior direction. During a typical 45-min treatment, fiducial seeds were tracked and adjustments to position were made at 30–60 s intervals. For each morning prior to SBRT, patients underwent a bowel prep including Dulcolax^®^ (Boehringer Ingelheim, Germany) and a Fleet^®^ Enema (C.B. Fleet Company, Inc., Lynchburg, VA, USA). In addition, at least 15–20 min before treatment, all patients received 1500 mg of amifostine (MedImmune, LLC Gaithersburg, MD, USA), mixed in saline and instilled into the rectum (13). The dose of radiotherapy consisted of either 35 or 36.25 Gy over five fractions, given daily for all patients. The initial 50 patients received 35 Gy. At that time, Stanford published on the feasibility of 36.25 Gy and we increased the dose for the next 30 months. After observing increased toxicity, we pulled the dose back to 35 Gy for the low-risk and low intermediate-risk (Gleason 6 with PSA >10, or Gleason 3 + 4 with PSA <10) patients.

For the homogenous planning, used in all patients, dose was normalized to the 83–87% isodose line in order for the prescription dose to cover at least 95% of the PTV. Generally speaking, dose volume histogram (DVH) goals for the rectum were such that the V50% <50% (i.e., the volume receiving 50% of the prescribed dose was <50%), V80% <20%, V90% <10%, and V100% <5%. The bladder DVH goals were V50% <40% and V100% <10%. For the bladder and the rectum, a typical D50 was 40–45% of the maximum dose. The femoral head DVH goal was V40% <5%. Urethra was not contoured as there was no urethral constraint.

### Follow up

The median follow up for the entire cohort was 72 months (0–96 months). In general, PSA values were obtained at baseline, and prospectively at 3 months post-treatment intervals during the first 2 years and at 6 months intervals thereafter. The PSA relapse definition used was the currently adopted standard of care Phoenix definition (i.e., nadir + 2) (14). bDFS was calculated with the Kaplan–Meier method and differences between groups determined by the log-rank test. A benign PSA bounce was called when PSA rose by >0.2 ng/mL above the post-treatment nadir and subsequently returned to nadir levels or below.

### Toxicity

Acute and late genitourinary (GU) and gastrointestinal (GI) toxicity was scored according to the criteria set forth by RTOG (15).

### Statistical analysis

The primary endpoint of the study was interval to bDFS. Kaplan–Meier survival method was used to estimate bDFS and log-rank *p*-values were used to compare the distributions. Cox regression analysis was used to significant risk factors. For Cox regression analysis, the assumption of the proportional hazards model was tested to ensure these assumptions were not violated. Two sided *p* < 0.05 was considered to be statistically significant. JMP Pro 10 (SAS Institute, Cary, NC, USA) was used for statistical analyses. For the multivariate analysis, categorical variables included were age (below vs. above median age of 68.6 years), hormones (no hormones vs. hormones), PSA (below vs. above 10 ng/mL), T-stage (T1 vs. T2), and Gleason score (Gleason 6, 3 + 4 vs. Gleason 4 + 3).

## Results

### Patient characteristics

Using this stratification, 324 patients were low-risk and 153 were intermediate-risk. Patient characteristics are summarized in Table 1. Median patient age at time of treatment was 68.9 (range 43.9–89.2 years old). 51 patients received up to 6 months of androgen deprivation therapy (ADT) prior to and during treatment, at the discretion of the urologist. The median PSA at diagnosis was 5.3 ng/mL. Fifty-nine patients have died, none from prostate cancer.

**Table 1:** **Patient characteristics at diagnosis**.

Age at diagnosis	Number of Patients	Percent	
Mean (range)	68.2		
Median	68.6 (43.8–89.2)		
40–49	4	0.8	
50–59	72	15.1	
60–69	190	39.8	
70–79	180	37.7	
80–89	31	6.5	
**PSA level at treatment**	**(ng/mL)**		
Combined mean (range)	6		
Median	5.3 (0.1–19)		

**PSA level at diagnosis**	**Number of patients**	**Percent**	***p*-Value**

<4 ng/mL	79	16.6	0.08
4–10 ng/mL	353	74.0	
>10–20 ng/mL	45	9.4	
**Risk Category**			
Low	324	67.9	0.015
Intermediate	153	32.1	
**Clinical Stage**			
T1a	2	0.4	<0.001
T1c	434	91	
T2a	41	8.6	
**Gleason Score**			
6	354	74.2	0.008
7 (3 + 4)	83	17.4	
7 (4 + 3)	40	8.4	
**Hormone Treatment**			
No	426	89.3	0.31
Yes	51	10.7	
**RT Treatment**			
35 Gy	154	32.3	0.098
36.25 Gy	323	67.7	

### Biochemical disease-free survival

**Figure 1:**
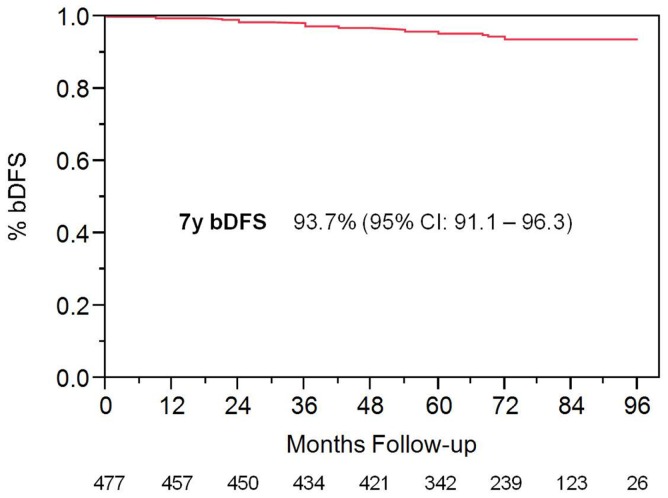
**Biochemimical disease-free survival in entire patient cohort**.

**Figure 2:**
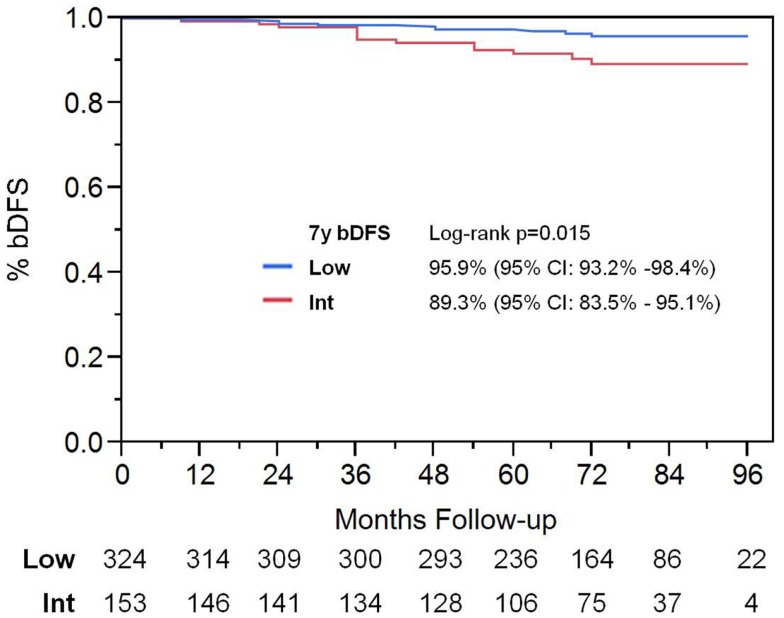
**Biochemical disease-free survival stratified by D’Amico risk group**.

With a median follow up of 72 months, the 7-year actuarial bDFS rate was 93.7% for all patients (Figure 1). It was 95.9 and 89.3% for low- and intermediate-risk patients, respectively (*p* = 0.015) (Figure 2). At last follow up, 59 patients had died, none from prostate cancer, with 7-year actuarial survival of 85%.

There were 123 patients possessing a minimum of 7 years follow up (range 84–96 months). For the first 50 patients treated, all of whom received 35 Gy, the 8-year bRFS is 97.9% with a median follow up of 96 months. Of this group, 41 patients had low-risk disease.

Most of the failures were distant, as local failure defined by positive biopsy was seen in only 0.9 and 2.6% of low- and intermediate-risk patients, respectively.

**Figure 3:**
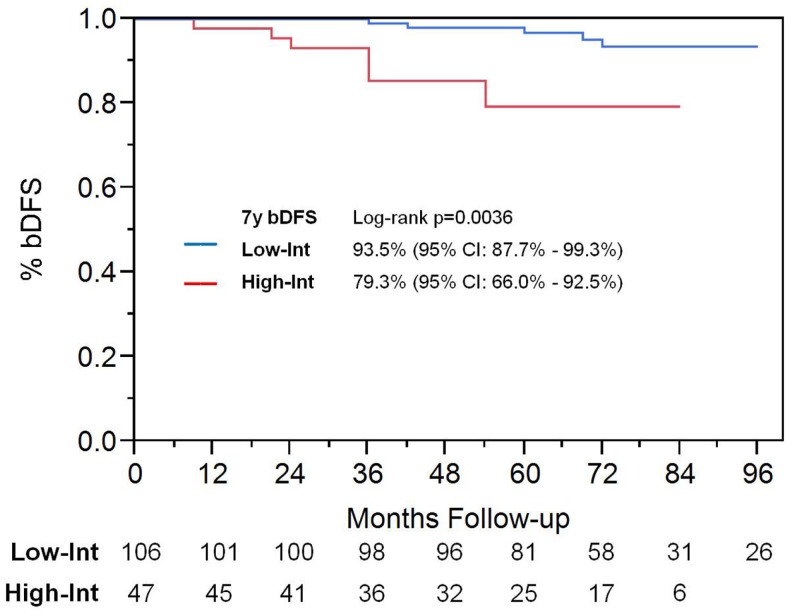
**Biochemical disease-free survival in intermediate-risk group patients**. Patients were stratified into low intermediate-risk and high intermediate-risk based on Gleason score and PSA. Patients deemed to be intermediate-risk due to one factor alone, of either Gleason score 3 + 4 or PSA 10–20, were considered to be intermediate-risk. Patients with Gleason 4 + 3 or 3 + 4 with PSA 10–20, were considered to be high intermediate-risk.

Among patients with intermediate-risk disease, those considered to have low intermediate-risk (Gleason 6 with PSA >10, or Gleason 3 + 4 with PSA <10; *n* = 106) had a significantly higher bDFS than patients with high intermediate-risk (Gleason 3 + 4 with PSA 10–20 or Gleason 4 + 3; *n* = 47) (Figure 3). Low intermediate-risk patients had a bDFS of 93.5 vs. 79.3% for high intermediate-risk patients (*p* = 0.0036).

**Figure 4:**
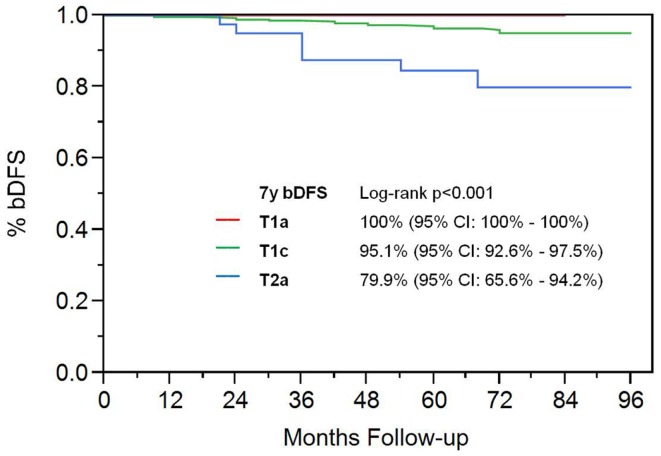
**Biochemical disease-free survival stratified by T-stage**.

**Figure 5:**
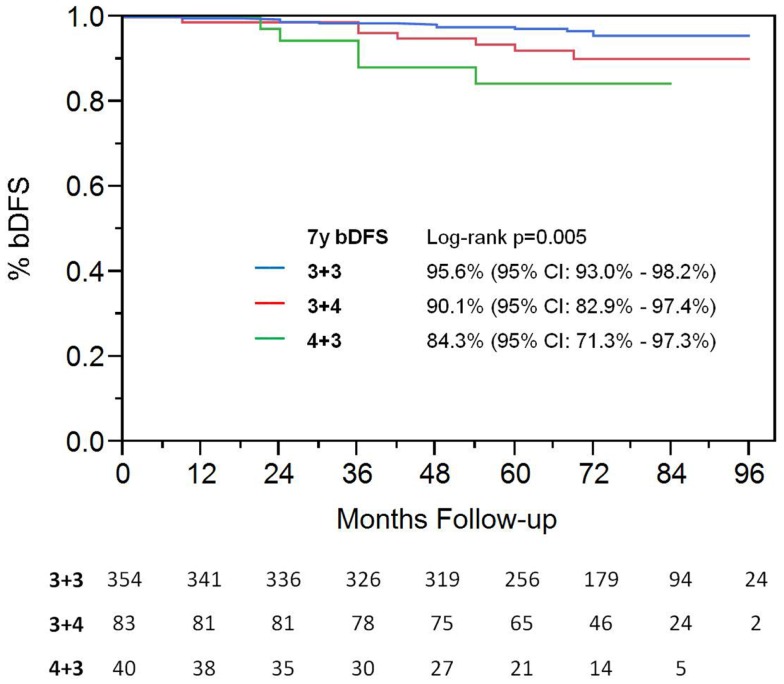
**Biochemical disease-free survival stratified by Gleason score**.

**Figure 6:**
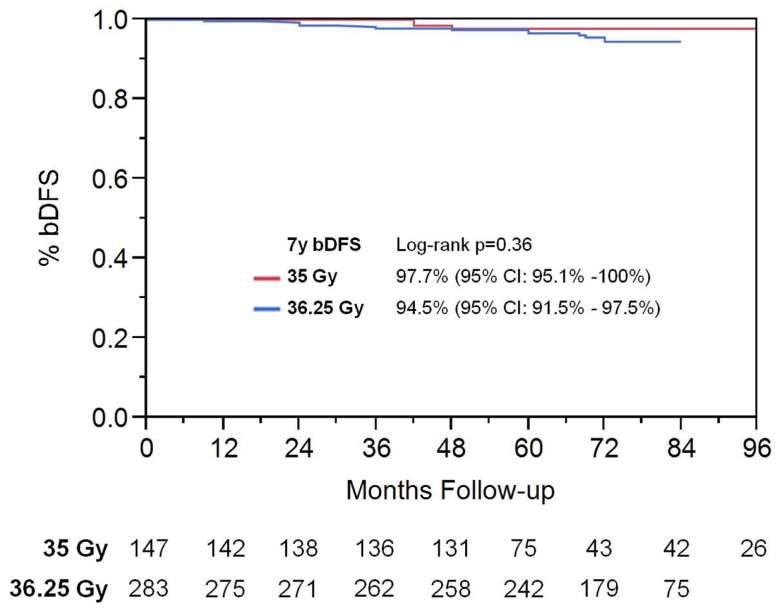
**Biochemical disease-free survival stratified by SBRT dose in low-risk and low intermediate-risk group patients**.

**Figure 7:**
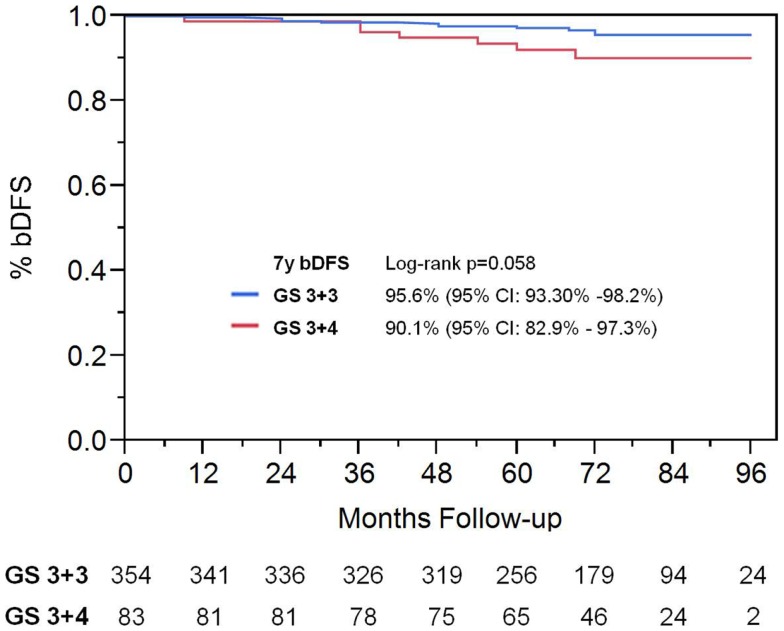
**Biochemical disease-free survival comparing patients with Gleason score 3 + 3 vs. Gleason score of 3 + 4**.

On univariate analysis, the addition of ADT was not a significant predictor of bDFS (Table 1). T-stage (*p* < 0.001) and Gleason score (*p* = 0.008) were significant predictors of outcome (Figures 4 and 5). Low-risk and low intermediate-risk group patients were stratified by dose (35 vs. 36.25 Gy, Figure 6), and dose was not significant for bDFS (*p* = 0.36), with 7y bDFS of 97.7 and 94.5%, respectively. There was no statistically significant difference between patients with Gleason score 3 + 3 = 6 and 3 + 4 = 7, though *p*-value trended toward significance (*p* = 0.058) (Figure 7).

### Multivariate analysis

Results of Cox multivariable regression analysis are shown in Table 2. Pretreatment risk factors analyzed included baseline PSA (above and below 10 ng/mL), clinical T-stage, use of ADT, age (above and below median), and Gleason score (3 + 3, 3 + 4, vs. 4 + 3). Variables found to be significant predictors for biochemical failure were T-stage (*p* = 0.0045, RR of 4.34) and PSA (*p* = 0.043, RR 3.21). Gleason score trended toward significance, with *p* = 0.073, RR 2.79.

**Table 2:** **Relative risk and *p*-value from Cox regression multivariable analysis for pretreatment predictors of biochemical failure**.

	*p-*Value	RR	95% CI	
Age (median 68.6)	0.44	1.38	0.60	3.26
T-stage (T1 vs. T2)	0.0045	4.34	1.64	10.35
PSA	0.043	3.21	1.039	8.27
Hormones (N vs. Y)	0.10	0.24	0.013	1.26
Gleason score (≤3 + 4 vs. 4 + 3)	0.073	2.79	0.89	7.23

*Categorical variables were age, T-stage, PSA (≥10 ng/mL), use of hormones, and Gleason score*.

### PSA trend

**Figure 8:**
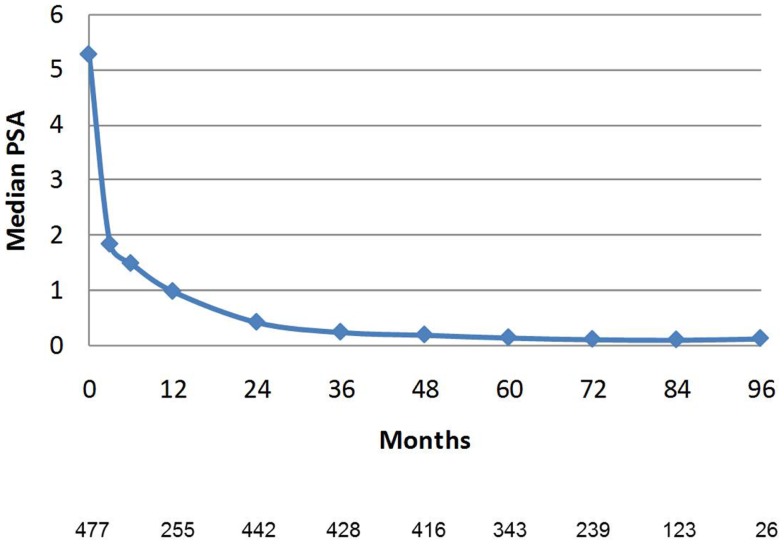
**Median PSA value in the entire patient cohort**. Error bars reflect interquartile range. The Number of patients with PSA data at each time point is listed below. Patients with biochemical recurrence are excluded.

**Figure 9:**
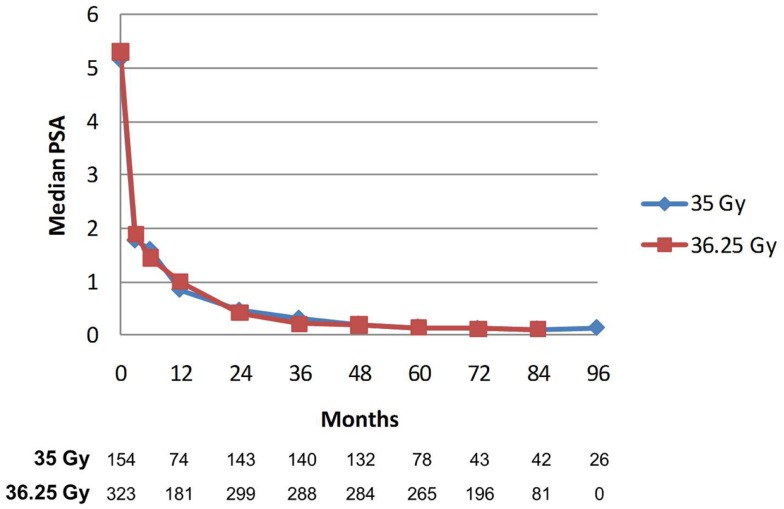
**Median PSA value stratified by dose**. There was no significant difference between median PSA values for any of the data points. The Number of patients with PSA data at each time point is listed below. Patients with biochemical recurrence are excluded.

PSA decline after SBRT gradually fell to an overall median of 0.11 ng/mL at 7 years (Figure 8). For the cohort of patients with 8-year follow up, median PSA remained low, at 0.11 ng/mL. Median time to nadir was 48 months (range, 3–84 months). A PSA bounce of >0.2 ng/mL was noted among 16% of patients at a median of 36 months (range, 3–60 months), with median bounce height of 0.50 ng/mL (range, 0.2–5.29). There was no statistically significant difference in bDFS for patients with PSA bounce vs. no bounce. Dose made no difference in the level of median PSAs at all measurement points (Figure 9).

### Toxicity

There was no grade 3–4 acute GI or GU toxicity observed. Late grade 3 GU toxicity (either retention requiring surgery or bleeding requiring laser coagulation) occurred in nine patients (1.7%). All grade 3 toxicity events occurred in the patient cohort that received 36.25 Gy. There was no late Grade 3–4 GI toxicity noted.

## Discussion

With the longest follow up of any published series using SBRT for prostate cancer thus far, there remains a very high rate of disease control in both low- and intermediate-risk patients. For both risk groups, results are similar to other hypofractionated dose schemes, such as HDR brachytherapy (16, 17). Compared to HDR brachytherapy, SBRT is non-invasive and can be performed as an outpatient procedure. SBRT may possibly have an advantage over IMRT as well. Zelefsky et al. published a series on IMRT with similar length of follow up (18). With 8-year median follow up, only 89 and 78% of low- and intermediate-risk patients were biochemically controlled, respectively, which is inferior to our results for SBRT. These results were corroborated by a series from Cleveland Clinic, demonstrating 85 and 70% local control with 81 Gy IMRT for low- and intermediate-risk patients, respectively (19). Radiobiology suggests SBRT may deliver a higher BED to the prostate, which may explain the discrepancy in local control between IMRT and SBRT. For example, 35 Gy in five fractions has an EQD of 90 Gy at 1.8 Gray fractions, using α/β ratio of 1.5. This higher EQD may explain why the bDFS rates for SBRT and HDR seem higher than IMRT with standard fractionation. Of course, mature data from randomized control trials are required to confirm this hypothesis.

After several years of follow up, PSA values drop to very low levels. As we see in our study, the median PSA is 0.4 ng/mL at 2 years and 0.2 ng/mL at 4 years. There are data suggesting that low PSA values predict durability of response (20). Consistent with this, our high biochemical control rates in short-term follow up translate to consistently high rates of biochemical control at long-term follow up of as long as 8 years. We predict there will be a continuation of biochemical disease control as patients are followed out beyond 10 years, as our present median PSA values remain quite low, at 0.1 ng/mL.

Our results continue to suggest that, even at longer follow up, there is no benefit in using higher dose SBRT. There is no significant difference in bDFS or median PSA. This finding can help guide SBRT dose selection, as 35 Gy appears to be just as effective as higher dose SBRT, at least for low- and intermediate-risk patients. We predict that increasing the SBRT dose above 35 Gy will result in increased toxicity without yielding improvement in outcomes; there are now reports suggesting this to be the case (21, 22). We hypothesize that this is due to a sigmoid dose–response curve for prostate cancer. There is a study from Jefferson University that analyzed over 10,000 patients using a variety of dose regimens, and demonstrated that a BED of 200 Gy maximized local control for prostate cancer, across all risk groups, because this was the beginning of the flattening of the dose–response curve (23). SBRT to 35 Gy is equivalent to 90 Gy at 1.8 Gy/fraction, or 200 BED (α/β of 1.5). Assuming these findings are true, then increasing SBRT dose above 35 Gy (and thus above BED of 200) will not provide any local control advantage.

Looking at the intermediate-risk group, a few observations of importance can be made. First, our results are comparable to reports on use of HDR brachytherapy as a boost after external beam therapy (16, 17). Thus, SBRT alone (without EBRT), may possibly be an excellent treatment for intermediate-risk disease. We have previously published data suggesting that, for high-risk disease, there is no benefit in performing EBRT + SBRT, compared to SBRT alone (24). Secondly, SBRT yields different biochemical disease control rates for the high intermediate-risk patients than for the low intermediate-risk patients. In fact, the low intermediate-risk group has a control rate that is similar to low-risk patients. We hypothesize that the decreased bDFS in the high intermediate-risk group is secondary to a greater propensity to develop distant metastatic disease, rather than a greater likelihood of local failure. If so, increasing the radiation dose to the prostate would not be expected to improve biochemical DFS. Our data also show no significant benefit for use of of ADT. As with high-risk patients, strategies such as use of adjuvant chemotherapy should be explored to try to reduce the risk of distant disease progression.

## Conclusion

CyberKnife SBRT produces excellent long-term biochemical control rates. Median PSA levels continue to compare favorably with other radiation modalities and with long-term follow up, results continue to demonstrate durability of response. It appears that 35 Gy is as effective as 36.25 Gy for low-risk and low intermediate-risk patients. Randomized trials will be necessary to prove whether there is an advantage over standard dose IMRT.

## Conflict of Interest Statement

The authors declare that the research was conducted in the absence of any commercial or financial relationships that could be construed as a potential conflict of interest.
